# Metal Doped PVA Films for Opto-Electronics-Optical and Electronic Properties, an Overview

**DOI:** 10.3390/molecules26102886

**Published:** 2021-05-13

**Authors:** Mircea Bulinski

**Affiliations:** Department of Theoretical Physics and Mathematics, Optics, Plasma and Lasers, Faculty of Physics, University of Bucharest, 077125 Bucharest, Romania; mircea_bulinsky@yahoo.com or mircea.bulinsky@unibuc.ro

**Keywords:** PVA, optoelectronic properties, metal doped polymers

## Abstract

Polyvinyl alcohol is unique among polymers. Apart from its preparation, it is not built up in polymerization reactions from monomers, unlike most vinyl-polymers, and it is biodegradable in the presence of suitably acclimated microorganisms. It is an environmentally friendly material for a wide range of applications, from medical ones, based on its biocompatibility, to integrated optics. This paper reviews, in addition to the preparation and optimization of films of polyvinyl alcohol doped with different metal species, the role of dopants and doping technologies in the involved electronic mechanism. The optical properties were studied by UV-VIS-IR, Mössbauer spectroscopy, and other measurement techniques, with applications such as real-time holography, microlasers, optical sensors or nanophotonics in mind.

## 1. Introduction

The unique and easily adaptable properties of polymers and polymer-composites open up possibilities for using these materials in an indefinite number of applications. Among various special features, the easy processing techniques and optical properties of novel compound polymers, makes them reliable candidates for various optoelectronic applications, in integrated optics, optical sensors, microlasers, nanophotonics, optical communication, and data processing [1,2,3,4,5].

The best known water-soluble polymer used for various optoelectronic devices and applications that is environmentally friendly and cost effective is definitely polyvinyl alcohol (PVA) [6,7,8], which is also one of the most important polymers used in the industry. With very good optical properties, PVA is a semicrystalline polymer, with high dielectric strength, high transparency and excellent physical properties [8]. Even if the best low light-scattering-loss polymer glasses (with the lowest refractive index, *n* = 1.3–1.4) for high transmission rate are amorphous polymers (perfluoropolymer-0.3 dB/km, at 1.3 μm, comparable with silica fiber) [9], PVA has been extensively analyzed due to its large number of possible applications relating to its chemical versatility [10] and its high optical characteristics. 

Among the many potential applications of doped PVA in high-tech photonics, we can list: highly diffractively efficient plates for holography or real time holography [11,12]; biocompatible microlasers (dye-doped PVA) based on polyvinyl alcohol microspheres [13]; white laser emission where the active material is composed of a solid polyvinyl alcohol matrix with a phase-separated liquid crystalline mixture [14]; LEDs, electroluminescence boards and plasma devices [15]; linear film polarizers in VIS domain (PVA sheets that are stretched and then dyed with iodine during the manufacturing process [16]), used in LCD displays or projection systems, and possibly even in UV domain [17]; wide-scale UV–VIS laser cut-off filters [18]; and so on.

**Figure 1:**
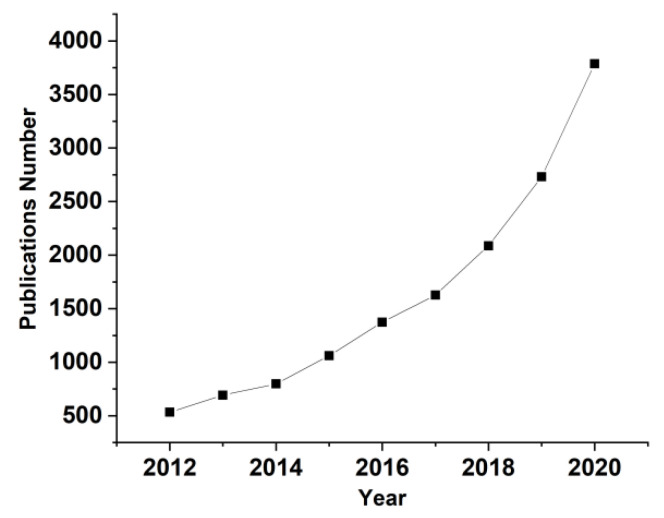
Annual number of publications about “Metals doped PVA” over the last decade in the fields of physical and chemical sciences (source of information: ReadCube- www.readcube.com, accessed on 30 March 2016).

Metals form an enormous number of inorganic compounds, and many of them are able to become a potential dopant/filler for polymers, including PVA in this case, at least for the purposes of scientific research on the properties of the resultant compounds. A simple and eloquent proof of interest in these types of composites is the graph of the annual number of publications about “metals doped PVA”, see Figure 1. This review is not intended to be exhaustive, but to outline some of the directions in which research is heading.

## 2. PVA-Synthesis and Properties

**Figure 2:**
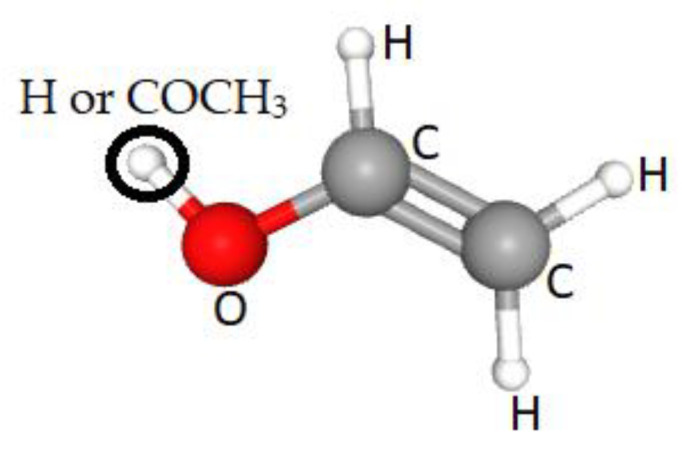
The PVA partially hydrolyzed monomeric structure (where **O** = H or COCH_3_).

PVA is a water-soluble polymer represented by the formula (C_2_H_4_O)_n_, see Figure 2, and is produced by the saponification of poly(vinyl ester) using sodium hydroxide (NaOH) [19], or by hydroxylation of polyvinyl acetate (PVAc). The vinyl alcohol (not used as the polymerization starting material because it is unstable and cannot be isolated) is hydrolyzed in methanol, ethanol, or a mixture of alcohol and methyl acetate, using alkalis or mineral acids as catalysts [20]. The resulting reaction removes the acetate groups from the PVAc molecules without breaking their long-chain structure, and when the reaction is complete the result is highly soluble in water and insoluble in practically all organic solvents (incomplete removal results in less solubility in water and more solubility in certain organic liquids) [21]. PVA occurs as an odorless, white-/cream-colored granular powder, with a commercially available degree of polymerization between 500 and 5000 (the number of monomeric units in a macromolecule equivalent to a molecular weight range of 20,000–200,000) [22]. PVA density is 1.19–1.31 gm/cm^3^; it has a melting point of 230 °C and decomposes rapidly above 200 °C as it can undergo pyrolysis at high temperatures [23]. The basic properties of PVA depend upon the degree of polymerization and the distribution of hydroxyl groups, as well as stereoregularity and crystallinity [24].

PVA is an atactic polymer (most atactic polymers do not crystallize, remaining in their amorphous state due to the absence of chain regularity) that can crystallize because its OH-groups are sufficiently small to not disturb the crystal lattice, and it has good optical transparency [25]. The refractive index of the PVA matrix is situated in the medium domain for polymers, *n* = 1.48–1.50 in the visible spectral range [26], and it has a high transparency, starting from approx. 40% at 200 nm (greater than 95% over 350 nm), and reaching almost 100% over 500 nm [27]. The refractive index of PVA is very useful in optics and photonics due to its ability to increase light output while reducing reflection loss.

### 2.1. PVA-Applications

PVA is a polymer with a wide variety of applications, used mainly as a component of adhesives and emulsifiers, as a water-soluble protective film, and as a starting material for the preparation of other resins [28] or polymeric nanoparticles. These can be obtained from most polymers in combination with PVA and Water-in-Oil-in-Water (W/O/W) by the emulsification solvent evaporation method, when, in most cases, poly(vinyl alcohol) is used as stabilizer of the emulsion [29].

Due to its ability to retain water (almost equal to that of natural cells) and its biocompatible, biodegradable, and nontoxic properties, it is one of the oldest and most extensively used synthetic polymer hydrogels, having several advanced biomedical applications such as wound management, organ drug delivery, artificial organs, and contact lenses [30,31]. The tensile potency of PVA resembles to that of human articular cartilage, its hydrophilicity and chemical stability allowing it to withstand extreme pHs and ambient temperatures, while its semicrystalline structure permits efficient oxygen and nutrients passage to cell [32].

In medicine and pharmacology, PVA fibers and films are used because of their ability to swell and absorb toxic products, decompose necrotic masses, and reduce blood loss [33]. They can be also used for coating SPIONs, and for the fabrication and development of magnetic particles for gene therapy [34]. It is shown that solutions containing up to 5% PVA are nontoxic to fish [35].

PVA, with its carbon–carbon backbone, being water soluble but not liable to hydrolysis [36], requires an oxidation process for its biodegradation and can be biodegraded by microorganisms in addition to enzymes through an oxidation or photo-oxidation process, under both aerobic and anaerobic conditions [37].

Many functional groups within the backbone enable PVA to be polar, and form hydrogen bonds that facilitate polymer-blend formation [38]. Morever, the hydrophilic character and the high density of reactive chemical functional groups helps PVA to be able to cross-link with dopant chemical materials [39].

Even if it is mostly used in food, medicine and cosmetics, PVA has proven to have numerous other applications, such as high quality thin film deposition, integrated optics and photographic films manufacture [40]. PVA is well known for its high dielectric strength compounds [41], thermal and chemical stability, and dopant-dependent physical properties [42].

### 2.2. PVA Films-Synthesis and Tailoring Properties

PVA is a hydrophilic polymer with excellent film forming properties as well as good processability and mechanical strength for film/membrane fabrication, but the major disadvantage of PVA based film for opto-electronic applications is its higher water absorption. To prevent such a problem, a possible solution is coating PVA film with protective layers to eliminate water absorption from the environment when no direct air/polymer interface is required; for example, triacetyl cellulose (TAC) film is used as a protective PVA based polarization layer in liquid crystal display (LCD) [43]. Other studies have reduced intrinsic polymer hydrophilicity. Using thermal crosslinking and adding different additives, an optimistic conclusion has been reached, namely that PVA has great potential for use in the synthesis of composites/films, as underlined in [44]. Table 1 shows the effects of PVA film treatments on the optical and mechanical properties of the final compound.

PVA thermally degrades at temperatures just exceeding 200 °C, at which temperature it often sees the onset of melting and crystallizes from the melt so rapidly that 100% amorphous samples cannot be obtained. In [59] it is shown from the heat capacity increment curve vs. mass fraction crystallinity (well fitted by the two-phase model comprising crystalline and mobile amorphous phases) that there is no evidence for the existence of a third phase, namely a rigid amorphous phase. In semicrystalline polymers (with two phases) crystalline and amorphous regions have no sharp boundary, allowing one macromolecule to run across the two regions. We can treat the PVA polymer as a crystalline lattice with voids filled by amorphous matter [60].

In general, PVA films with no special treatments have a degree of crystallization of approx. 30–40% [48], which can be increased gradually only by annealing the films, to 60% (at a maximum of 150 °C, where, most likely, one sees the chemical destruction of the polymer, the hydroxyls are lost, and polyene and other chemical structures are formed [45]). When adding different dopants, like 0.1 mol/L lithium chloride, the crystallization is reduced to 18% [45], or with chitosan (to 15%), and with supplementary glycerol down to 1% [48].

A highly crystallized molecular structure of final PVA films generates a polymer with low mechanical strength and water resistance performance [46]. Pure PVA is semi-crystalline, and the degree of crystallinity can be controlled by the film/gel method, by polymer blend with a plasticizer (resins, rubber and elastomers) [48,61,62], or even by dopant insertion. For example, the lower crystallinity of the PVA films with the addition of various conductive ions may be due to the strong interaction among the polymer molecules and conductive ions, as with Ni^2+^ [47], or ferric ions Fe^3+^, when the crystallinity of PVA is reduced from 41.6 to 7.7% with the addition of 2.0% ions [46].

The presence of acetate groups affects the ability of PVA to crystallize upon heat treatment [63], which is of particular interest for crosslinked hydrogels. The crystalline degree of PVA can be improved by repeated cycles of freezing and thawing, and by gamma ray irradiation [56]. An alternative method to control crystallinity is the use of derivate polymers, such as acrylamide (a carcinogenic compound) modified PVA, prepared by the alcoholysis of vinyl acetate and acrylamide copolymers. This enhances the water solubility and tunes the tacticity (with effects on the physical properties of the polymer) and crystallinity [64].

It is known that aqueous solutions of PVA gradually undergo gelation upon standing at room temperature (from the formation of networks in which the PVA crystallites generated by spinodal decomposition serve as the junction points) [65]. For gel formation through freezing/thawing methods, as the number of cycles is increased, the number and stability of crystallites also increases (due to the condensation of the PVA solution by the formation of ice) [66]. Low-temperature crystallization is the most popular method used to prepare PVA gel, with excellent mechanical properties, but it uses toxic dimethylsulfoxide (DMSO) as a solvent [67]. A simpler and more eco-friendly method for preparing transparent PVA hydrogel with an enhanced crystallinity, with water as the only solvent, uses swollen PVA placed into a hot-pressing machine (95 °C), pressed from 2 to 20 MPa (in successive steps from 2 to 15 min), and kept at room temperature without drying for one week (for gelation) followed by drying in air for two days, followed by vacuum drying for 2 days. The gel thus prepared shows a decrease in net crystal size and good mechanical properties [68].

Other methods to improve the specific properties of PVA for different applications involve blending it with other polymers: with poly(vinylpyrrolidone) PVP [49,50,69] for increased thermal, electrical, mechanical stability and electrochemical properties; with polyethylene glycol (PEG) for facilitated gas transport [70], with PVAm in order to improve film formation and mechanical properties; reinforcing it with carbon nanotubes [52] or with poly(glucosyloxyethyl methacrylate) (poly(GEMA)) for the suppression of the thermal decomposition of PVA in an aqueous solution, and to increase the thermal decomposition temperature above 300 °C [51].

### 2.3. Preparation of Doped PVA Films

Commercially available PVAs have a high level of versatility and therefore ability to be adjusted to specific needs. They are characterized by a wide range of polymerization/hydrolysis depths, molecular weights (expressed in different solution viscosities), and a variable degree of removal of the acetate groups from the raw material (polyvinyl acetate).

The preparation of doped PVA films for optoelectronics applications with certain physico-chemical characteristics can only be analyzed in the complex context of the process as a whole, because any change in each stage can significantly alter the final product [71,72]. In general, the phases of preparation start with the initial PVA powder (with a certain molecular weight and degree of hydrolysis and polymerization), which is dissolved in solvent (usually simple mono-distilled water or deuterium oxide, water methanol mixture, acetonitrile, dimethyl sulfoxide (DMSO), dimethylformamide (DMF)/water, chloroform, etc.) by ultrasonication, stirred or placed in water at 60–90 °C for one to three hours (time and conditions vary according to the molar weight and the degree of hydrolysis), followed by doping; then it is placed in an oven at the same temperature for a further couple of hours to eliminate air bubbles. Other steps may be taken, depending on the nature of the doping or blending/mixing with other polymers or substances. The gel/solution is deposited onto support plates (casting, dip coating, spin coating or dipping into the substrate, etc.) with controlled thickness (for more details, see [71,73,74,75,76]). In the final treatment of the film it is dried in heat or in a vacuum, and irradiated.

For thick films a crystalline skin is formatted [77] that consists of a glassy layer formation on top of the evaporating solution at the air–sample interface, because the solvent removal rate is faster compared to the “bulk” solution system [78].

One of the simplest methods for improving PVA films is gel ultrasonication, through which the film gains larger lattice strain and a more crystal structure [54]. The use of an ultrasonic probe with a power greater than 300 W for up to 10 min increases the tensile strength by almost 30%, decreases the strain at break by 30% and the water vapor permeability by 11%, but unfortunately opacity also decreases by 22%.

For some applications, the process of deposition can be repeated to obtain a multi-layered structure, with each layer having a different thickness or physico-chemical characteristics. In [79] a few-atoms-thick layers of transition metal carbides were assembled into a compact network for conducting heat and electrons. PVA can also be manufactured into microspheres, having a size range of 10 to 250 μm (with 1 to 5 μm wall thickness), by droplet technique [80] or by the self-assembly (due to surface tension) of dispersed micro-droplets of dye-doped PVA solution. These are injected into uncured polydimethylsiloxane, heated at 80 °C for 90 min, and resin removed by a solvent; obtaining solid-state dehydrate microspheres for microsphere biolasers [13].

Sometimes the organometallic PVA films can be used as an intermediate material to obtain other structures used in optoelectronics. Deposed on a conventional polymer or composite, when cured, the organometallic polymer decomposes to form a highly dispersed nanoscale metal oxide phase that gives a high refractive index (up to 1.9 depending on the metal oxide content) to the final coating (see [81]).

Obtaining nanoparticles and nanowires can be also mediated by PVA film, as using a capping agent (to regulate growth and final particle size) in the process of clustering nanocomponents and aqueous-based synthesis is known as one of the most popular methods to prepare nanoparticles [82,83,84]. In [84] binary (NiO and Cr_2_O_3_) nanoparticles were synthesized by thermal treatment method. Nickel and chromium acetate are added as metal precursors to PVA aqueous solution, mixed into a homogeneous solution, and cast and dried at 80 °C for 24 h. The ground powder is calcined at 500–800 °C for 3 h (this treatment is usually called the solution combustion method). Similarly, ternary metal oxide nanocomposites were synthesized from Zn and Fe nitrate, or Mn sulphate [85].

## 3. Tuning the Optical and Electronic Properties of Doped PVA Films

Due to the structure of PVA and the chemical specificity of additives, it is easy to change the physical characteristics of the polymer film, whose properties can be improved by introducing different dopants/filers into the polymer matrix, such as salts, carbide, nanocomposites, ions, or even other polymers. Table 2 briefly shows the effects of some metal dopants on PVA films.

The properties of the obtained composites depend drastically on the modifier’s nature. Two quaternary ammonium salts, with similar composition, are used as polymer additive, Cloisite15A and Cloisite30B [55]. The first is slightly intercalated by the interacting polymer macromolecules (during melt compounding) and contributes to the enhancing of the glass transition temperature and the mechanical properties of the resulting composites. On the contrary, the second, which is significantly intercalated, disturbs to some extent the H-bonding network established within the polymeric matrix, thus showing a reduction in thermal and mechanical properties.

The performance of many solid-state devices (optical sensors, integrated optical circuits, emissive displays, etc.) can often be improved by applying a transparent high refractive index coating onto the light-emitting/sensing surface of the device (a gradual transition from the high refractive index of the active circuitry to the low index of air allows light to be coupled more effectively). Blending PVA with other polymers is a way of obtaining such a film, like in [53], where a biodegradable composite film of PVA and PVP shows an increase in the PVA refractive index of up to 20%; another way is to dope PVA.

Metal ion implantation (and plasma treatment) is used for the alteration of the physico-chemical and electro-mechanical properties of different materials, to improve the optical and conductive properties of ceramic materials. For inert polymers, an ion beam may produce ultra-thin conducting or reflecting films below the polymer surface. Among others, this technique has been applied for strain gauge applications [98] or for PVA, for increasing the degree of crystallinity and microhardness [57], for fabricating planar light guides [99], and for enhanced photoluminescence [58].

In [98] X-ray photoelectron spectroscopy reveals that after ion beam treatment of PVA, the metallic implant (magnesium and zinc in this case) is in both metallic and oxidized states, and surface morphology modification (depending on the implantation dose only) denotes an increase in free surface energy, the degree of crystallinity, and microhardness. In [99], PVA films implanted with Li^+^ and N^+^ ions exhibit an anisotropy in refractive index. The variations upon irradiation were found to be larger than 0.05 in PVA, which are interpreted as being generated by the formation of aromatic compounds in the regions of electronic scattering. Analyzed with m-line spectroscopy, a 3–5 μm thickness film (guiding light up to three modes) irradiated with a low implantation dose (<10^14^ ions/cm^2^) leads to a total waveguide loss values lower than 2 dB/cm (for 633 nm wavelength ). A cellulose reinforced polyvinyl alcohol silica (Cel-PVA-Si) composite implanted using N^3+^ ions [58], shows significantly modified luminescent, thermal and mechanical properties, also as an electric conductivity improvement by about 25%. FTIR analysis reveals that the ion implanted samples undergo bond breakage. The formation of microstructures, which improve the surface roughness, increase the stiffness of the sample by about 50 times compared to that of the unaltered films. The photoluminescence of the native cellulose is improved greatly by defect site and dangling bonds.

PVA doped with metal inorganic compounds - PVA shows good compatibility with most inorganic/organic fillers, and enhanced composites may be prepared without the need to introduce coupling agents and interfacial modifiers [100].

**Figure 3:**
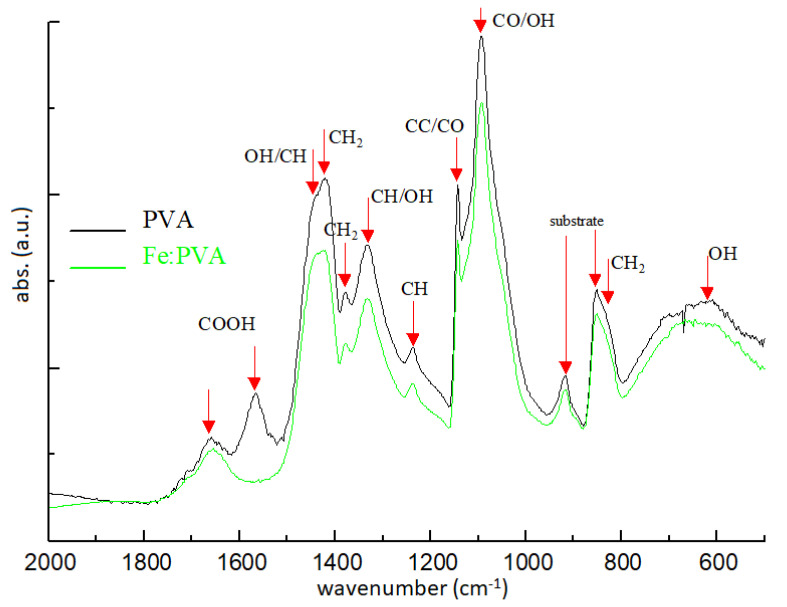
IR absorption spectra (FTIR at 4 cm^−1^ resolution) for PVA (MW 14,000-completely hydrolyzed) and Fe:PVA (5 wt% FeCl_3_).

Polymers doped with transition metals were an early solution to the growing interest in optical data processing technologies in the 1990s. Metal oxides and inorganic salts were mainly used as additives in vinyl-polymer solutions, but almost any inorganic metallic compound can play this role. In the IR absorption spectra of PVA and PVA:FeCl_3_ doped film (Figure 3) the significant spectral bands of the polymer constituents can be observed compared to the spectrum of the doped polymer.

**Figure 4:**
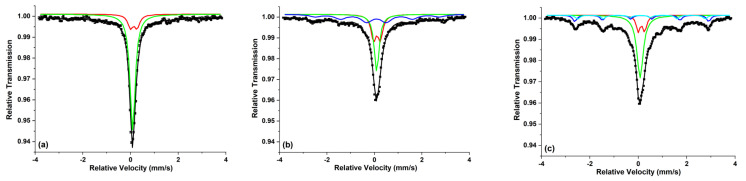
The 4.2 K ^57^Fe Mössbauer spectra of the Fe:PVA (2 wt% FeCl^3^) films with: (**a**) 5 wt% PVA, (**b**) 8 wt% PVA, (**c**) 11 wt% PVA.

Even if the doping of an aqueous polymer is performed with metal salt solutions, where the dopant metal is assumed to be a simple chemical compound, the final distribution of metal atoms in polymer is not always of the same size nor of the same complexity. For example the main species in aqueous solutions of ferric chloride are the octahedral complex [FeCl_2_(H_2_O)_4_]^+^ and the tetrahedral [FeCl_4_]^−^, the latter is predominant in solutions with high chloride concentrations and at high temperatures [101]. The final film of doped Fe:PVA, obtained from the mixture of both salt and polymer solutions, contains small groups of Fe ions due to the particular PVA intimate semi-crystalline structure. A direct proof of the aggregation of Fe ions into small clusters of 1–2 nm size (<100 atoms) [92] is the specific behavior of the temperature dependent Mössbauer spectra of PVA films doped with FeCl_3_. While the Mössbauer spectra (see Figure 4) of single molecules of FeCl^3^ in PVA should consist of paramagnetic doublets until the lowest temperature of 5 K, the presence of a magnetic spectral component (sextet) at 5 K [102] gives evidence for the presence of small clusters of Fe^3+^ ions with a blocking temperature lower than 50 K. In fact, in many cases the metal compound forms clusters [103] and so it is always difficult to distinguish the polymers doped with metals from those doped with nanoparticles [104].

Doping can be used only to modify the mechanical or crystallinity characteristics of PVA films. To lower the crystallinity degree of the PVA films we can add various conductive ions; this is due to the strong interactions between the polymer molecules and conductive ions, as with Ni^2+^ [47] or ferric ions, when the crystallinity of PVA is reduced from 41.6% to 7.7% with the addition of 2.0% ions [46], and the tensile strength of the modified PVA membrane is increased by 240%. Moreover, with a tougher structure and improved fluidity, the strength of ferric ion modified PVA bonded samples is increased by 157% [46].

Out of the polymers doped with transition metals, ferric chloride doped polyvinyl alcohol (Fe:PVA) was analyzed with respect to changes in the optical properties under UV exposure [86], and its possible applications to holography and integrated optics. The decrease in the refractive index and the increase of the absorption coefficient in the visible range, induced by UV exposure, are related to the reduced oxidation state of the doping metal (Fe^3+^ →Fe^2+^) and to the redistribution of the electronic density around the doping metal without changing its valence. In the Mössbauer spectra obtained at room temperature, excepting the central paramagnetic patterns assigned to Fe^3+^ ions, doublets with higher quadrupole splitting and isomer shifts (namely those typical for Fe^2+^ ions) appear to rise in intensity with the UV exposure. The diffraction efficiency experimental data were satisfactorily explained in correlation with the Fe^2+^/Fe^3+^ ratio [87].

Erbium chloride (ErCl_3_) is a rare-earth metal chloride salt which was analyzed in terms of its optical properties in interaction with PVA [88], with possible applications in optoelectronics. Each dispersed complex molecule of rare earth is a luminescent unit, and the luminescent behavior of the erbium–polymer composites is determined by interfacial interactions between the polymer matrix and the luminescent species. When Er^3+^ is mixed with a host PVA, it induces the mixing of states, leading to new transitions in the erbium atom due to the specific electronic behavior of Er^3+^ (the partially filled 4f shell is shielded by filled 5s and 5p shell) [88]. Prepared by being casted from their aqueous solutions, PVA:ErCl_3_ (doped with 9 wt% of ErCl_3_) exhibits an energy gap of 5.1 eV and has a refractive index of 1.72. The major change in the energy gap with the concentration of erbium (0.5 eV) was attributed to the complexes’ formation between Er^3+^ ions and OH groups of PVA structure.

Titanium chloride (TiCl_3_) is another doping salt of PVA, for which the optical properties of compound polymeric films were examined by optical absorption and emission spectroscopy [105]; it can play a role in the modification of the optical and dielectric properties of PVA in order to make it more applicable. The complex determination of electrical properties was also performed, related to the electrical conductivity at room temperature for different concentrations of TiCl_3_ (in 20 Hz to 3 MHz domain). The dielectric constant indicates a strong dielectric dispersion and increases with dopant concentration, and the optical energy gap decreases dramatically as TiCl_3_ increases up to a doping level of 10%, and then remains constant [105].

Similarly to metal chlorides, mono-elemental chloride salt metal-doped PVA was prepared for multiple valence metals as Antimony (Sb) and Tin (Sn), for which the UV exposure induces significant valence changes of the metal ion, mainly for polymers containing residual water, while the electron transfer phenomena depend strongly on the doping metal [106]. The list of chloride salt metals which have been used as PVA dopant is very large, see for example Vanadium (V) [107], Barium (Ba) [108] and Copper [Cu] [109].

Metals can be embedded in PVA, like in the case of Cobalt (Co), ultrasonically mixed with the solution of PVA, obtaining metal-doped PVA films [95]. The complete dried films, obtained by the casting method, were analyzed by X-ray diffraction, scanning electron microscopy, FTIR, and UV–Vis spectra in 190–900 nm domain. Different optical parameters such as energy bandgap and dielectric loss are calculated from the transmission and absorbance measurements. The optical limiting performance of PVA/Co-metal films was measured at the wavelengths of 632.8 and 533 nm. X-ray diffraction confirms that the effect of the fillers’ concentration on the crystallinity of the polymer material is related to an increase in internal strain and a decrease in particle size (the growth of distortion and imperfection due to the strong interaction between the PVA matrix and the metal—here, Cobalt). The peaks observed from the Co-metal increased with the concentration of Cobalt in polymer, and the intensity of diffraction peaks from PVA decreased, probably due to interaction between the Co-particles and the hydrogen bond. In film Co/PVA FTIR spectra, the PVA matrix feature dominates at a small Co-metal percentage, and the decrease in the intensity of the functional vibration groups in the matrix reflects the strong interactions between the metal and the PVA. In the UV–Vis spectra, the transmittance gradually declined, due to the adding of Co-metal causing the particles to agglomerate and more incident light to be absorbed or reflected, reaching less than 8% for PVA/10% Co films. The induced absorption bands indicate an energy-band gap reduced from 5.33 eV to 4.61 eV between the π and π* bonding and antibonding molecular orbitals, as a result of adding Co-particles at a ratio of 0.1–10% in the matrix of PVA. The energy gap has a significant role in optoelectronic construction and solar cells, and as a whole the polymeric composite is appropriate for laser attenuation and optical limiting in photonic devices.

**Figure 5:**
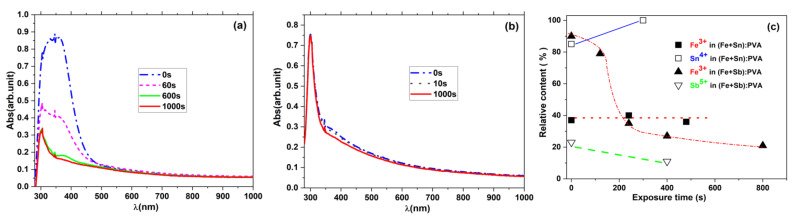
Optical absorption spectra of (**a**) (FeCl_3_ (2.5%) + SbCl_3_ (2.5%)): PVA (10%); (**b**) (FeCl_3_ (2.5%) + SnCl_2_ (2.5%)): PVA (10%) samples after different UV exposures, and (**c**) the variation of the relative fraction of metallic ions in the highest oxidation state in the corresponding samples.

PVA doped with multi-element metal compounds - Related to mono-elemental metal-doped PVA, simultaneous doping with two or more metals is another possibility for improving the desired properties of the materials for optoelectronics. PVA doped with pairs of metal ions shows a strong dependence of its optical properties on the electronic configuration inside the composite [110]. A comparative study between PVA films doped with multiple valence metal pairs of (Fe + Sn) and (Fe + Sb) and mono-elemental Fe, Sn and Sb [11] points to the correlation between the evolution of the optical absorption in the UV range and the corresponding local electronic phenomena induced by UV exposure (see for example Figure 5). This induces a variation of the refractive index, with possible applications for real time holography. Analyzed by ^57^Fe, ^119^Sn and ^121^Sb Mössbauer spectroscopy, under UV irradiation, experiments show that the Fe ionic state reduces, the Sn oxidizes (proving an inverse electron transfer compared with the case of iron) and Sb stabilizes in its lower Sb^3+^ valence state and exhibits no charge transfer phenomena [106]. After exposure of the Fe–Sn pairs, the Sn^2+^ species are transformed completely while the Sn^4+^ and Fe^2+^ species remain almost unchanged, and for the Fe–Sb pairs the relative proportion of the Fe^2+^ components increases rapidly, while Sb^5+^ decreases (the overall percentage of the Sn^4+^ and Sb^5+^ species changes by less than 10%, while F^3+^ changes by 70%), showing that the main influence on absorption is the charge transfer phenomena involving the transition Fe^3+^→Fe^2+^ [110].

Many other combinations of metals pairs are possible, for example PVA doped with a CrO^3^+CuO pair induces changes in the morphology of the polymer films and in the electronic charge transfer processes, with direct influences on the optical properties of polymer films, transmittance and refractive index [92].

PVA metal nanocomposite-Nanomaterials have been studied extensively in the last decade due to their wide applications and innovative possibilities. During nanocomposite formation, the agglomeration of nanofillers commonly takes place due to their high surface energies. A uniform dispersion of nanofiller in a polymer matrix is a key factor in the enhancement of the nanocomposites, and this is in many cases the role of PVA.

Trying to analyze the effects of multi-metal polymer doping in [96], it was shown that combinations of Zn, Mg, and Cd formed doped PbS/PVA freestanding nanocomposite films. Pb(NO_3_)_2_ and PVA in deionized water generated the initial Pb^2+^/PVA solution, in which various concentrations of metal ions including Zn(NO_3_)_2_, MgSO_4_, and Cd(NO_3_)_2_ were directly added. The preparation process was completed by adding a drop of Na_2_S (forming the metal-doped PbS/PVA nanocomposite), before it was casted, left to dry, and peeled off from the substrate to obtain 80 μm freestanding films. From the absorbance spectra of PbS/PVA, Zn-PbS/PVA, Mg-PbS/PVA, and Cd-PbS/PVA, the calculated optical band gap (2.44 eV. for PbS/PVA) increased with increased dopant molar ratio for all prepared samples. The nature of the nonlinear refraction and the performances of the all-optical limiting of the films were investigated using the modified Z-scan technique [96] with a CW laser of 532 nm wavelength. Results showed an increase of less than a factor of two for the limiting threshold only between PbS/PVA and Zn-PbS/PVA doped with 0.008 M Zn, at a limiting threshold of 3 mW with an optical damage of 21 mW for a high dynamic range of 7.

In [94], nanocomposite films of cerium oxide (CeO_2_)-doped PVA were prepared by a solution casting technique using nanoparticles synthesized by a solution combustion method. The X-ray characterization showed the size of the nanoparticles in the composites to be 6.5–44 nm. The UV-VIS spectrum of the composites exhibited a red shift in the optical band gap with respect to pure PVA and both red and blue shifts with respect to nano-CeO_2,_ depending on the concentration of the filler. Good absorption in the ultraviolet and visible ranges suggest possible use in filters and solar cells. The measurement of the photoluminescence (with an excitation wavelength of 325 nm; 3.8 eV) reveals three emission peaks corresponding to the UV and blue–green regions of the visible range, and a decrease in intensity at higher concentrations (maybe due to the process of self-absorption of light). The maximum of photoluminescence intensity (nearly equal to that of nano CeO_2_) was observed for a doping concentration of 2.5 wt%, suggesting that this concentration is most suitable for optoelectronic applications [94]. When dielectric materials like metals-doped polymers are placed under an applied electric field, they manifest electrical conduction and polarization phenomena, characterized by a dielectric constant, an important parameter in optoelectronic devices. Intending to control them, in [93] separate zinc oxide (ZnO) and copper oxide (CuO) nanofillers of PVA based composite were investigated using solution casting technique. The electrical properties of composite strips (AC conductivity, dielectric constant and dielectric loss) were evaluated using a frequency response analyzer. [93] observed a rise in the electrical properties in ZnO but a decrease in CuO–PVA composite strips, and the frequency response analysis showed a prominent effect, suitable for use in MHz range microelectronics and microwave applications. In another study, PVA containing copper sulphide nanoparticles (CuS) is analyzed using x-ray diffraction, FTIR, a scanning electron microscope, and UV-VIS [89]. Incorporated CuS nanoparticles present a crystalline nature. Their conductivity (increasing with dopant concentration as well as with frequency) and dielectric behavior were studied over the frequency range of 300 Hz to 1 MHz in the temperature range of 30–110 °C. The absorbance of nanocomposite samples increased, and the optical band gap energy decreased with increasing CuS concentration. The optical and electrical performances of PVA can be enhanced by the addition of a small amount of CuS nanoparticles (0.04 M), and the material can be adapted for optoelectronic devices [89]. In [91], the dielectric and conductivity behaviors of nano-ZnO-doped polyvinyl alcohol composites (for various concentrations of dopant) were also investigated, and a significant increase in dc and ac conductivities was found, making them a potential candidate for applications in electronic devices. Co-doped ZnO nanowires embedded in a PVA matrix [90] improve the bandgap and have a good photoluminescence, with thermal properties superior to that of polymer matrixes. The material may have applications in visible light-emitting diodes, nanophotonic devices, UV-shielding sunglasses, window glass, and photocatalytic materials for pollutant degradation.

Other classes of new metal compounds with elaborated chemical structures, nanomaterials like MXene or quantum dots/crystals, etc., can be used as filer/dopant materials for PVA. PVA has qualities such as low optical absorption in VIS-NIR region, good thermal properties, low cost, and lightfastness, showing an optimal combination of both systems’ useful features.

MXene (few-atoms-thick layers of transition metal carbides, nitrides, or carbonitrides) are another class of inorganic compounds used for doping PVA with applications in optoelectronics [79,97]. A 2D nanomaterials/polymer composite-based film is presented in [97] for all-optical modulation (broadband and ultrafast optics). Starting from a MAXene crystal (Ti_3_AlC_2_), a nanosheet dispersion of Ti_3_C_2_T_x_ (with layered structure) is prepared and mixed with a PVA host polymer, then dripped and dried to obtain a thin film. The composite film is inserted into the middle of two fiber connectors’ sandwich structure to characterize the MXene nanosheets, polarization-dependence and photothermal effect. The structure presents a linearly increasing light power/temperature slope of 1.02 °C/mW, a polarization-dependent broadband intensity modulation with a depth of 15 dB, and a rising(falling) time of 238(330) μs, a fast response time among fiber-typed all-optical modulators based on the thermo-optic effect [79].

## 4. Conclusions

PVA (Iodine-doped) is very well known for its industrial application as a high-quality optical polarizer in the UV and VIS regions as much as for its varied applications in different industrial markets. Valuable in new high-tech applications, its utility in optoelectronics has been an important subject for scientific analysis, and the metal doped PVA remain an opened research field. Mono, paired or multi-element metal compounds have been tried for tuning the final material to a specific set of properties, and the results are promising, indeed.

## Figures and Tables

**Table 1:** Effects of PVA film treatments on optical and mechanical properties.

Treatment	Parameter Values: {up}-Increasing; {dw}-Decreasing	Refs.
Adding dopants (conductive ions)	{dw}: Degree of crystallinity, tensile strength	[45,46,47]
Blending (plasticizers, polymers)	{up}: Thermal stability, electrical, mechanical, and electrochemical properties	[48,49,50]
Heat treatment (freezing and thawing)	{dw}: Degree of crystallinity	[31]
Reinforced with poly(GEMA)	{up}: Thermal decomposition temperature	[51]
Reinforced with carbon nanotube	{up}: Mechanical properties	[52]
Blending with biodegradable PVP	{up}: Refractive index	[53]
Ultrasonic	{up}: Tensile strength. {dw}: Water vapor permeability, strain at break	[54]
Polymer additive	{up}: Glass transition temperature, mechanical properties	[55]
Gamma ray irradiation	{dw}: Degree of crystallinity	[56]
Ion beam	{up}: Reflecting coefficient, degree of crystallinity, photoluminescence	[57,58]

**Table 2:** Effect of different dopants on optical and electrical properties of PVA.

Dopant	{up}-Increasing; {dw}-Decreasing	Application Areas	Refs.
FeCl_3_	{up}: VIS absorption. {dw}: Refractive index	Real time holography	[86,87]
ErCl3	{up}: Refractive index. {dw}: Band gap.	Optoelectronics	[88]
CuS	{up}: Electrical conductivity {dw}: Band gap, dielectric constant, dielectric loss	Optoelectronics	[89]
Co-ZnO	{up}: Photoluminescence, Thermal stability. {dw}: Band gap.	UV-shielding, nanophotonics	[90]
ZnO	{up}: AC conductivity, dielectric constant, Tensile strength, elongation at break. {dw}: Band gap, dielectric loss.	Optoelectronics, EMI, and UV shielding, microwave absorption, UV luminescence	[91]
CrO_3_+CuO	{dw}: Refractive index, high transmittance	Sensor applications	[92]
CuO	{dw}: Band gap, dielectric constant, dielectric loss, AC conductivity	Optoelectronics	[93]
CeO_2_	{up}: Absorption in the UV; Photoluminescence (UV, blue–green)	Filters; Solar Cells	[94]
Co-metal	{up}: Absorption. {dw}: Energy gap	Optical limiting in photonic devices	[95]
Zn-PbS	{dw}: Band gap, optical nonlinearity, dynamic range	All-optical limiting	[96]
MXene	{up}: Broad spectrum, optical modulation	Polarization-dependent all-optical modulator	[97]
